# Induction of cell death by pyropheophorbide‐*α* methyl ester‐mediated photodynamic therapy in lung cancer A549 cells

**DOI:** 10.1002/cam4.1012

**Published:** 2017-02-09

**Authors:** Ping‐hua Tu, Wen‐jun Huang, Zhan‐ling Wu, Qing‐zhen Peng, Zhi‐bin Xie, Ji Bao, Ming‐hua Zhong

**Affiliations:** ^1^ Department of respiratory medicineThe Central Hospital of XiaoganXiaoganChina

**Keywords:** A549 cells, apoptosis, cell cycle, photodynamic therapy, pyropheophorbide‐*α* methyl ester, reactive oxygen species

## Abstract

Pyropheophorbide‐*α* methyl ester (MPPa) was a promising photosensitizer with stable chemical structure, strong absorption, higher tissue selectivity and longer activation wavelengths. The present study investigated the effect of MPPa‐mediated photodynamic treatment on lung cancer A549 cells as well as the underlying mechanisms. Cell Counting Kit‐8 was employed for cell viability assessment. Reactive oxygen species levels were determined by fluorescence microscopy and flow cytometry. Cell morphology was evaluated by Hoechst staining and transmission electron microscopy. Mitochondrial membrane potential, cellular apoptosis and cell cycle distribution were evaluated flow‐cytometrically. The protein levels of apoptotic effectors were examined by Western blot. We found that the photocytotoxicity of MPPa showed both drug‐ and light‐ dose dependent characteristics in A549 cells. Additionally, MPPa‐PDT caused cell apoptosis by reducing mitochondrial membrane potential, increasing reactive oxygen species (ROS) production, inducing caspase‐9/caspase‐3 signaling activation as well as cell cycle arrest at G_0_/G_1_ phase. These results suggested that MPPa‐PDT mainly kills cells by apoptotic mechanisms, with overt curative effects, indicating that MPPa should be considered a potent photosensitizer for lung carcinoma treatment.

## Introduction

Lung carcinoma constitutes the most commonly encountered malignancy worldwide, and the prime killer among all cancers. Non‐small cell lung cancer (NSCLC) amounts to about 80–85% of pulmonary carcinoma cases 1. The majority of patients are diagnosed with locally advanced or even metastatic disease, and unfortunately most of them will die as a consequence of the incurable illness 2. In recent years, surgery combined with adjunct chemotherapy has markedly increased patient survival rates; however, the overall 5‐year survival rate remains intriguingly low 3. Photodynamic therapy (PDT) achieves targeted therapy of solid tumors through local photo‐radiation of tumor cells after photosensitizer uptake, producing reactive oxygen species (ROS) and inhibiting cancer growth 4. PDT has been applied in multiple malignancies such as melanoma as well as head and neck, bladder, breast, and pulmonary carcinomas 5, 6, 7, 8. This approach has benefits of limited invasion and reduced toxic effects. However, ideal photosensitizers with better efficacy and less side effects yet to be developed. MPPa is a second‐generation photosensitizer derived from chlorophyll. This new derivative exhibits stable chemical structure, strong absorption, less normal tissue phototoxicity and longer activation wavelengths 9. The A549 cell is typical cell line as nonsmall cell lung carcinoma, researchers have explored photodynamic efficacy for different photosensitizers in A549 cells and clarify the mechanisms. This study aims to explore the effect of MPPa‐mediated photodynamic therapy on human lung cancer A549 cells in vitro and elucidate its possible molecular mechanisms.

## Materials and Methods

### Cell culture and reagents

A549 cells were obtained from the Institute of Radiation Medicine, Peking Union Medical College (China), and cultured in RPMI‐1640 containing 10% fetal bovine serum (FBS) and antibiotics. The cells were incubated at 37°C in a humid environment with 5% CO_2_. The above cell culture reagents were purchased from Gibco (Grand Island, USA). MPPa, Cell Counting Kit‐8, 2′,7′‐dichlorofluorescin diacetate and Hoechst 33342 were obtained from Sigma‐Aldrich. Annexin V/PI double staining and JC‐1 mitochondrial membrane potential detection kits were manufactured by Keygen Biotech (Nanjing, China).

Rabbit monoclonal antibodies against human caspase‐3 and ‐9, Bcl‐2, and Bax, respectively, were manufactured by Cell Signaling Technology (Danvers, MA). Anti‐*β*‐actin and anti‐cytochrome‐c primary antibodies as well as secondary antibodies were purchased from Abcam (Cambridge, UK). The PDT equipment was manufactured by Chongqing Jingyu Laser Technology Co. Ltd. (Chongqing, China).

### Photodynamic treatment

The photosensitizer MPPa in DMSO (1 mmol/L) was filtered and sterilized. MPPa treatment was administrated for 20 h incubation in the dark. A semiconductor laser (630 nm) was employed as light source in PDT, at 40 mW/cm^2^. Light exposure was regulated by irradiation time, with five levels of 0, 1.2, 2.4, 4.8, and 9.6 J/cm^2^, obtained with illumination times of 0, 30, 60, 120, and 240 sec, respectively. The detail steps were just as we described in our previous study 10.

### Cell viability assessment

Cells were seeded into 96‐well plates at 1 × 10^3^ cells/well, and cultured in 100 *μ*L medium per well for 24 h to achieve cell attachment. Cells were treated with various test articles for 20 h. Afterwards, 10 *μ*L CCK‐8 was added per well for another 4 h. Absorbance was obtained on a microtiter plate reader at 450 nm; data were presented as mean ± standard deviation (SD). All experiments were carried out in triplicate. Then the cell viability was calculated according to the following formulation: cell viability (%) = OD_expriment_/OD_control _× 100%. Finally, MPPa at 1 *μ*mol/L and light dose of 4.8 J/cm^2^ were selected for subsequent experiment.

### Measurement of ROS production

Cells were treated in 24‐well plates (5 × 10^4^ cells/well, 1 mL). Afterward, 200 *μ*L DCFH‐DA staining solution at 10 *μ*mol/L was added to the cells for 20 min at 37°C in the dark. After careful removal of the medium and a washing step, ROS level assessment was carried out by fluorescent microscopy and flow cytometry.

### Hoechst nuclear staining

After treatment of A549 cells with MPPa‐PDT, staining was performed with Hoechst 33342 at 37°C (10 min). A fluorescent microscope with UV excitation was employed for analyses. Untreated cells served as a control group.

### Transmission electron microscopy

A549 cells were fixed in pellets (4°C) using 2.5% glutaraldehyde and 1% osmium tetroxide; this was followed by dehydration with grade ethanol and acetone, before embedding and sectioning. The specimens were then stained using uranyl acetate and lead citrate, and examined by transmission electron microscopy.

### Flow cytometry for apoptosis assessment

Cells (1 × 10^4 ^cells/well, 1 mL) were cultured overnight for attachment, and treated with 1 *μ*mol/L of MPPa in combination with 4.8 J/cm^2^ PDT for 24 h. All cells were harvested from each group for AnnexinV/PI double staining and analyzed flow‐cytometrically.

### Mitochondrial membrane potential (MMP) evaluation

Cells were treated in a 6‐well plate with 1 *μ*mol/L of MPPa in combination with 4.8 J/cm^2^ PDT for 3 h. The MMP was assessed flow‐cytometrically after JC‐1 staining as previously depicted 10.

### Cell cycle distribution

Cell fixation (70% ice‐cold ethyl alcohol) was performed at 4°C overnight. After centrifugation and washing, the PI staining solution was incubated with the samples for 30 min at 4°C in the dark. Finally, cell cycle distribution was assessed on a BD Facscalibur flow cytometer.

### Western blot

Treated A549 cells were lysed in cell lysis buffer at 4°C for 10 min. Equal amounts of total protein in lysates were resolved by SDS‐PAGE and electro‐transferred onto PVDF membranes. After blocking with 5% skim milk, anti‐caspase‐3 (1:1000), anti‐caspase‐9 (1:1000), anti‐cytochrome‐c (1:1500), anti‐Bcl‐2 (1:1500), anti‐Bax (1:1000) and anti‐*β*‐actin (1:2500) primary antibodies were added overnight at 4°C. This was followed by secondary antibody addition (room temperature, 1 h). Detection was carried out with the ECL Plus kit, and membranes were exposed to the G:BOX iChemi XR gel documentation system. The gray values of western bands were analyzed quantitatively by Quantity One software.

### Statistical analyses

The data were presented as mean ± standard deviation (SD), and assessed with the GraphPad Prism6 software. Group comparison was performed by one‐ or two‐way analysis of variance (ANOVA) for intergroup evaluation, and SNK‐q test for intragroup assessment. *P* < 0.05 was deemed to indicate statistical significance.

## Results

### MPPa‐PDT inhibited the viability of A549 cells

**Figure 1:**
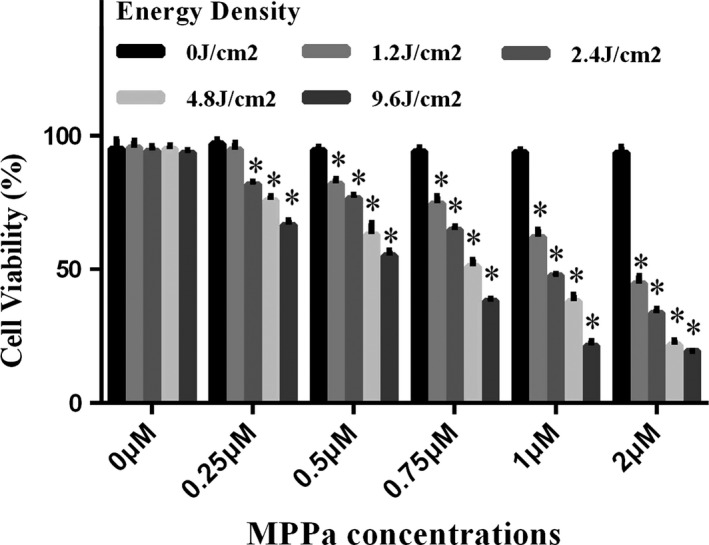
Cell viability was measured 24 h after PDT. A549 cells were incubated with various concentrations (0–2 *μ*mol/L) of MPPa for 20 h. Sensitized cells were irradiated with various LED (0–9.6 J/cm^2^). Data were presented as mean ± SD (*n* = 3). *P < 0.05 versus the control group.

The viability of A549 cells after MPPa‐PDT treatment was assessed, and data were shown in Figure 1. Single LED and MPPa groups did not significantly inhibit A549 cells (*P* > 0.05). The amounts of viable cells decreased with increasing light dose and MPPa concentrations (*P* < 0.05), except for those treated with 0.25 *μ*mol/L MPPa combined with 1.2 J/cm^2^ light dose (*P* > 0.05). In general, MPPa‐PDT showed a significant inhibition of A549 cell viability in a light‐ and drug‐dose‐dependent manner. Meanwhile, at light exposure of 4.8 J/cm^2^, half‐maximal inhibitory concentration of MPPa was 0.97 ± 0.05 *μ*mol/L. Treatment with 1 *μ*mol/L MPPa and light exposure at 4.8 J/cm^2^ resulted in 48.8%±1.9% cell death. Therefore, the optimal experiment condition (1 *μ*mol/L MPPa combined with 4.8 J/cm^2^ light dose) was selected for subsequent experiments exploring the molecular mechanisms of cell death in A549 cells.

### MPPa‐PDT induced ROS generation in A549 cells

**Figure 2:**
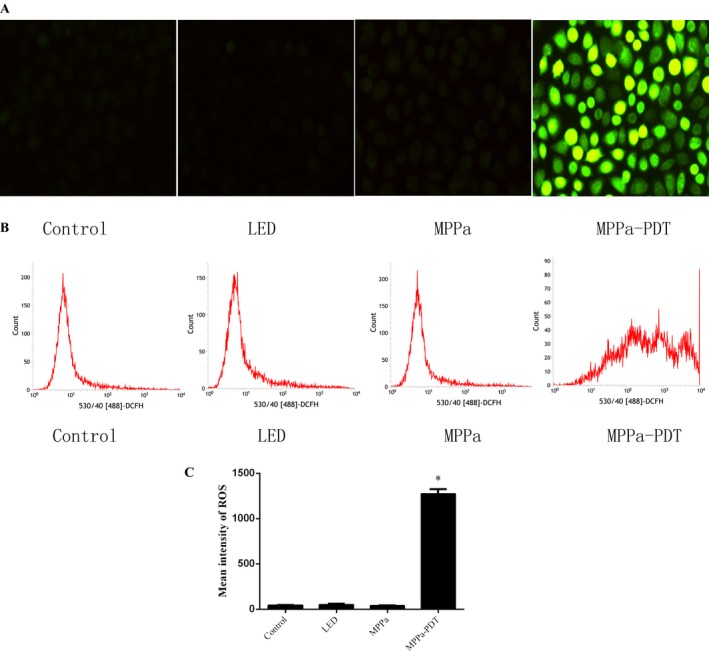
ROS production was induced by MPPa‐PDT. (A and B) Intracellular ROS generation in A549 cells detected by fluorescent imaging and flow cytometry using DCFH‐DA probe 3 h posttreatment.(C) Quantitative analysis of ROS levels (*n* = 3). *P < 0.05 versus the control group. ROS, reactive oxygen species.

Large amounts of ROS production were triggered after MPPa‐PDT treatment. Intracellular ROS generation in A549 cells were detected by fluorescent imaging and flow cytometry using DCFH‐DA probe after different treatments. After 20 h incubation of A549 cells with MPPa in the dark, they were irradiated; then, the ROS probe was added for assessing ROS amounts. No detectable ROS production in MPPa alone or LED alone groups was observed. Meanwhile, ROS levels were clearly elevated in the MPPa‐PDT group, as evaluated by fluorescence microscopy (× 200) and flow cytometry (Fig. 2A and B).

### MPPa‐PDT induced ultrastructural changes in A549 cells

**Figure 3:**
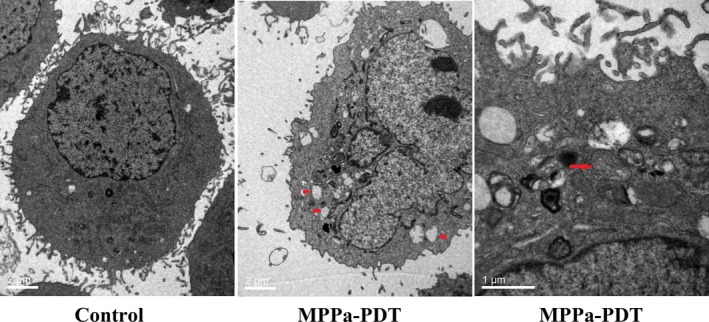
Transmission electron micrograph of A549 control cells and cells treated with MPPa‐PDT for 24 h. The treated cells lost their pseudopodia and exhibited cell shrinking, intracytoplasmic vacuoles. (arrowhead, magnification, × 6000), and mitochondria swelling (arrow, magnification, ×12000).

MPPa‐PDT‐treated A549 cells were assessed by transmission electron microscopy (TEM), which showed ultrastructural change. The shape of cells was altered and pseudopodia structure was damaged after MPPa‐PDT treatment. Meanwhile, intracytoplasmic vacuolation and mitochondrial swelling were seen (Fig. 3).

### MPPa‐PDT increased apoptosis of A549 cells

**Figure 4:**
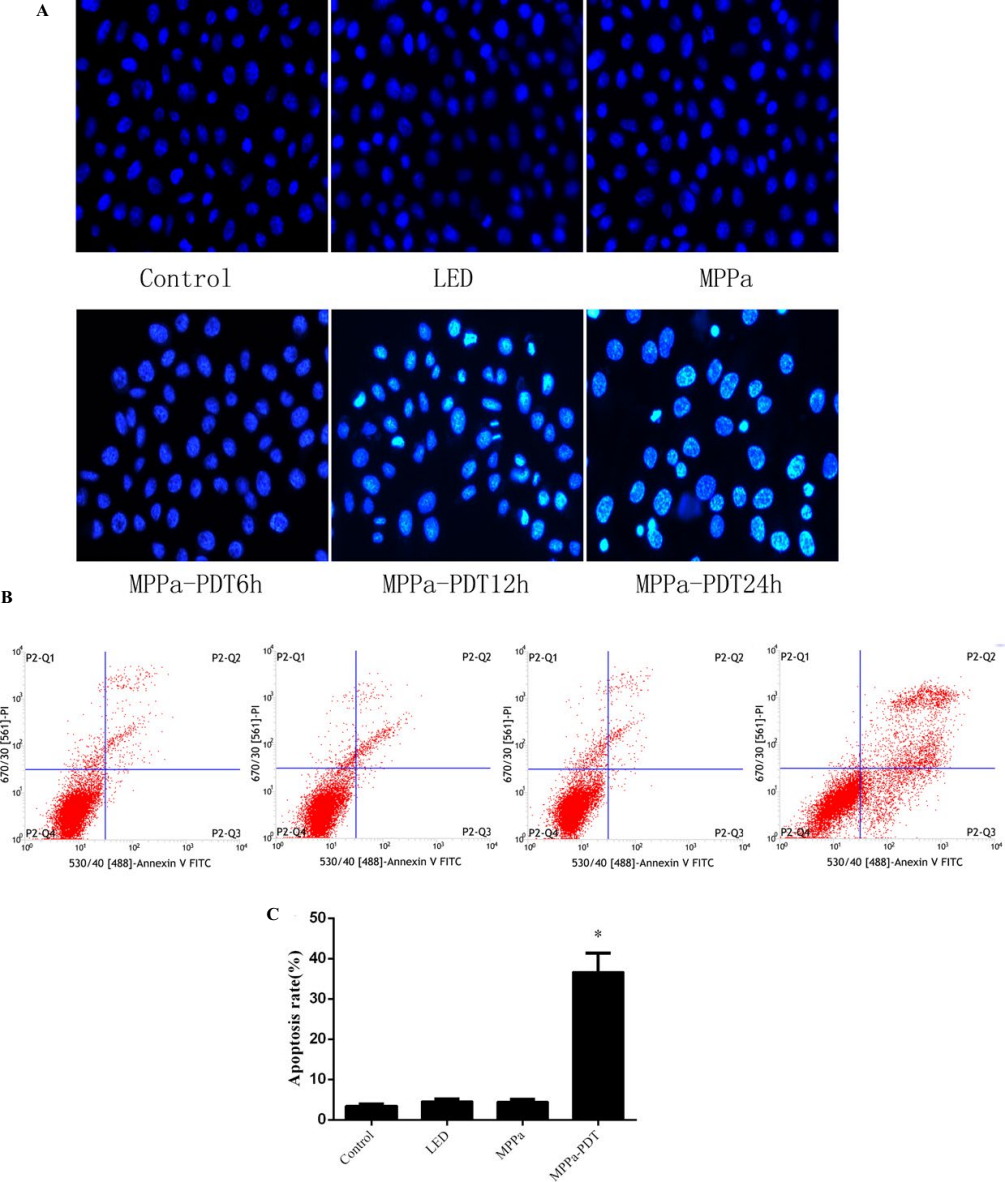
(A) Cell apoptosis observed by Hoechst 33342 staining. Apoptotic cells exhibited chromatin condensation and nuclear fragmentation. (B) MPPa‐PDT induced apoptosis in A549 cells as assayed by Annexin V/PI staining. (C) Quantitative analysis of apoptotic cells from B in a bar chart. *P < 0.05 versus the control group.

To verify whether MPPa‐PDT induced cell death in an apoptotic way, Hoechst staining and AnnexinV/PI staining were used in our experiment. After Hoechst staining, more apoptotic cells were found in the MPPa‐PDT group. Such cells had condensed chromatin, karyopyknosis, and nuclear fragmentation, as well as typical apoptotic bodies (Fig. 4A). Apoptosis was quantified by AnnexinV/PI staining (Fig. 4B). Interestingly, significantly higher rates of early and late apoptotic cells were obtained in comparison with the control, LED and MPPa groups, respectively (*P* < 0.05).

### MPPa‐PDT suppressed mitochondrial membrane potential in A549 cells

**Figure 5:**
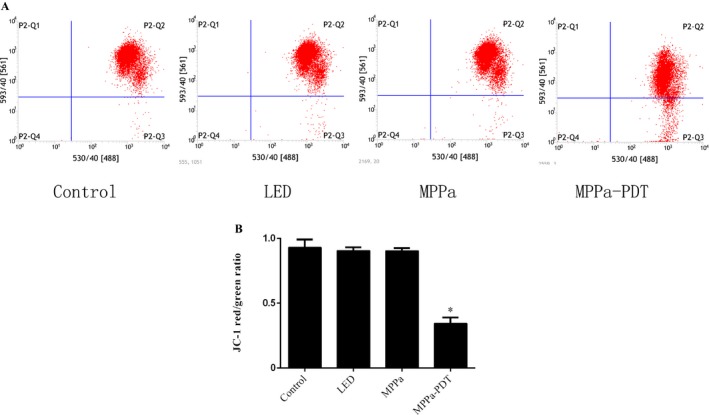
Mitochondria membrane potential was determined by JC‐1 fluorescent dye using flow cytometry analysis. (A) A549 cells were treated with MPPa‐PDT for 3 h. (B) The quantitative analysis of mitochondria membrane potential. Each sample was expressed as red/greeen fluorescence ratio (*n* = 3) *P < 0.05 versus the control group.

Several studies reported that the MPPa localized in mitochondria and mitochondrial photodamage contributed to photosensitizer‐induced programmed cell death. To further verify whether mitochondria involved in MPPa‐mediated photodamage, the mitochondrial membrane potential was examined by JC‐1 staining using flow cytometric analysis, as described above. Upon A549 cell treatment with MPPa in combination with irradiation, MMP was significantly reduced (Fig. 5A and B).

### MPPa‐PDT induced cell cycle arrest at G_0_/G_1_ phase

**Figure 6:**
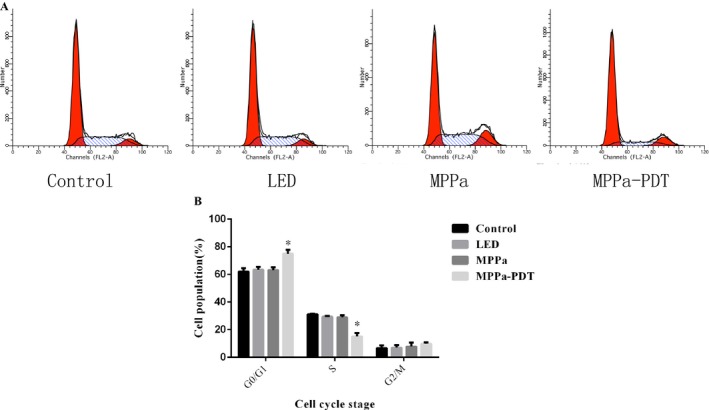
The cell cycle distribution was analyzed by flow cytometry. (A) A549 cells were treated with MPPa‐PDT for 24 h. (B) Percentage of cells at different phase was calculated in histograms. Data were presented as mean ± SD (*n* = 3). *P < 0.05 versus the control group.

To better understand the antineoplastic activities of photodynamic therapy, cell cycle arrest was performed as well. As shown in Figure 6, MPPa‐PDT induced G_0_/G_1_ cell cycle arrest in A549 cells. In comparison with the control group, percentages of G_0_/G_1_ phase cells in LED alone and MPPa alone groups were not significantly different. However, MPPa‐PDT treatment resulted in markedly higher amounts of G_0_/G_1_ phase cells, compared with the control, LED and MPPa groups, respectively. These findings indicated that MPPa‐PDT treatment resulted in enhanced G_0_/G_1_ cell cycle arrest.

### Mitochondrial pathway was involved in apoptosis induced by MPPa‐PDT

**Figure 7:**
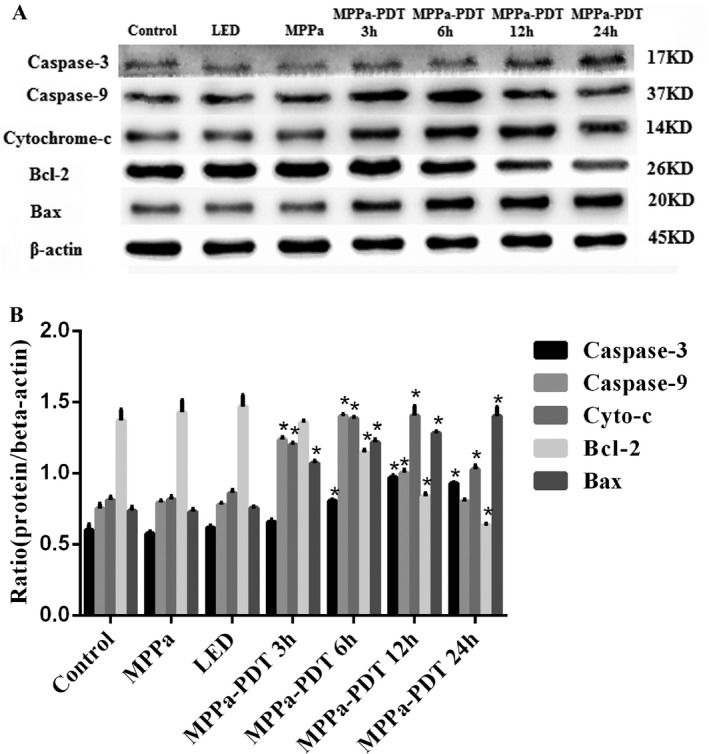
The expression of mitochondrial apoptosis pathway‐related proteins were assessed by western blot. (A) Effect of MPPa‐PDT on the expression level of caspase‐3, caspase‐9, cyt c, Bcl‐2, and Bax. (B) Quantitative analysis of protein expression level. Data were presented as mean ± SD (*n* = 3).*P < 0.05 versus the control group.

Mitochondrial damage could induce cytochrome c (cyt c) release into the cytoplasm. The cyt c activated Apaf1 to recruit caspase‐9 and subsequently induced caspase‐3 to trigger the apoptotic cascade. The proteins expression of mitochondrial apoptosis pathway was assessed by Western blot to explore the possible molecular mechanisms of MPPa‐mediated cell death. In this study, cyt c release coupled with caspase‐9 gradually increased and peaked in the cytosol after treatment with MPPa‐PDT for 6 h. In addition, the expression of caspase‐3 was also increased following MPPa‐PDT treatment. In addition, we found that Bcl‐2 expression decreased, with Bax gradually increasing after treatment with MPPa‐PDT. Our data demonstrated that MPPa‐PDT induced apoptosis mostly through mitochondrial caspase‐9/caspase‐3 signaling (Fig .7A and B).

## Discussion

PDT represents a therapeutic approach widely employed for treating various cancers 11, 12, 13. Its mechanism of action depends on combining a photosensitizer (PS), light source, and oxygen for tumor cell inhibition. PS is activated at an appropriate wavelength, leading to the generation of highly reactive singlet oxygen. These reactive oxygen species are involved in cytoplasmic organoid damage and promote tumor cell death. Meanwhile, PDT affects many cellular mechanisms, and can effectively avoid drug tolerance. MPPa‐mediated PDT has been studied in several tumor cells 10, 14. MPPa is a potent photosensitizer for both one‐ and two‐photon activated PDT, with potential applications for cisplatin‐resistant carcinomas 15. Tian et al. found that MPPa‐PDT kills prostate cancer cells mostly through apoptosis 16. Here, MPPa‐PDT applicability in the treatment of NSCLC was assessed.

As shown above, no significant cytotoxicity of single‐light radiation or MPPa administration was found in A549 cells. However, MPPa‐PDT showed a significant inhibition of A549 cell viability in a light‐ and drug‐dose‐dependent manner. Precisely, the optimal experiment condition was obtained for 1 *μ*mol/L MPPa used in combination with 4.8 J/cm^2^ light exposure.

ROS has a significant function in tumor cell treatment using PDT; excessive ROS levels lead to programmed cell death 17. The interaction of light with the photosensitizer causes ROS generation. This study demonstrated that there was no ROS production in the MPPa alone and single‐light radiation groups. Treatment with MPPa combined with irradiation, however, resulted in sharply increased ROS amounts. Chromatin condensation, karyopyknosis, nuclear fragmentation, as well as typical apoptotic bodies were found after MPPa‐PDT treatment. Meanwhile, MPPa‐PDT treatment resulted in cell shrinking, pseudopodia disappearance, multi‐vacuolation and mitochondria swelling, as demonstrated by electron microscopy. Quantitative apoptotic analysis of A549 cells treated with MPPa‐PDT was evaluated by flow cytometry. Our results showed that cell apoptosis rate in the MPPa‐PDT group was markedly increased compared with the values obtained for other groups. These findings indicated that inhibition of cell viability after MPPa‐PDT treatment occurred mainly via apoptosis in A549 cells. Mitochondrial dysfunction contributes to photosensitizer‐induced programmed cell death, playing a critical role in this pathway. In this study, the MMP was assessed by JC‐1 staining flow‐cytometrically, and obviously decreased in the MPPa‐PDT group, indicating that mitochondria‐dependent apoptotic cell death might be enhanced by MPPa‐PDT. To further assess whether the mitochondrial apoptotic pathway was affected by MPPa‐PDT treatment, cytochrome c release in the cytoplasm as well as caspase‐9 and ‐3 were evaluated.

Mitochondrial dysfunction causes MMP decrease and the release of cyt c, which activates Apaf1 to recruit caspase‐9 and subsequently induces caspase‐3 for programmed cell death initiation 18, 19, 20. In this study, cyt c release coupled with caspase‐9 gradually increased and peaked in the cytosol after treatment with MPPa‐PDT for 6 h. In addition, the expression of caspase‐3 was also increased following MPPa‐PDT treatment.

The Bcl‐2/Bax protein complex is an important regulatory factor for maintaining normal mitochondrial membrane permeability 21. Its expression can regulate cyt c release and activate the downstream caspase‐3 protease, which in turn mediates cell survival or death 22. We found that Bcl‐2 expression decreased, with Bax gradually increasing after treatment with MPPa‐PDT. These findings suggested that MPPa‐PDT might initiate apoptosis by regulating the Bcl‐2/Bax complex and further activating the mitochondrial caspase‐9/caspase‐3 pathway.

The antineoplastic activities of photodynamic therapy are generally attributed to cell cycle arrest and programmed cell death induction. Shao et al. reported that human hepatocellular carcinoma HepG2 cells treated with the photosensitizer photocyanine combined with irradiation mainly accumulated at the G_2_/M stage 23. Meanwhile, MPPa‐PDT induced PC‐3 cell cytotoxicity by causing cell cycle arrest at the G_0_/G_1_ phase 24. As shown above, following MPPa‐PDT treatment, cell amounts in the G_0_/G_1_ phase were increased and compared with control group values. These results were inconsistent with those of a previous study demonstrating that MPPa‐PDT stopped cell cycle progression from the more sensitive G_0_/G_1_ phase in the PC‐3M cell line. The discrepancy might be due to the fact that cell cycle distribution was associated with cell and photosensitizer types.

Overall, the current findings indicated that MPPa‐PDT‐induced cell apoptosis occurred mainly through caspase‐9/caspase‐3 signaling as well as cell cycle arrest at G_0_/G_1_. This study showed overt cytotoxic effects for MPPa‐PDT treatment, suggesting MPPa to be a potent photosensitizer for photodynamic treatment of lung carcinoma.

## Conflict of Interests

The authors have no conflicts of interest to declare in association with this study.
